# Supplementary material for: Single-particle cryo-EM structures from iDPC–STEM at near-atomic resolution
Source: Nat Methods. 2022 Sep 5;19(9):1126–36. doi: 10.1038/s41592-022-01586-0 (PMC9467914; doi:10.1038/s41592-022-01586-0)
Supplement: Supplementary file 1 — Supplementary Tables 1–3 and Figs. 1–6. [file 41592_2022_1586_MOESM1_ESM.pdf]

nature portfolio

https://doi.org/10.1038/s41592-022-01586-0

Supplementary information

Single-particle cryo-EM structures from iDPC–STEM at near-atomic resolution

In the format provided by the
authors and unedited

Supplementary Material

Single-particle cryo-EM structures from iDPC-STEM at near-atomic resolution

Authors: Ivan Lazić[1]*, Maarten Wirix[1], Max Leo Leidl[2,3,4,5], Felix de Haas[1], Daniel Mann[2,5], Maximilian Beckers[2,6], Evgeniya V. Pechnikova[1], Knut Müller-Caspary[3,4], Ricardo Egoavil[1], Eric G.T. Bosch[1], Carsten Sachse[2, 5, 7, *]

[1] Materials and Structural Analysis Division, Thermo Fisher Scientific, Eindhoven, The Netherlands.
[2] Ernst Ruska-Centre for Microscopy and Spectroscopy with Electrons (ER-C-3): Structural Biology, Jülich, Germany.
[3] Department of Chemistry and Centre for NanoScience, Ludwig-Maximilians-University Munich, Munich, Germany
[4] Ernst Ruska-Centre for Microscopy and Spectroscopy with Electrons (ER-C-1): Physics of Nanoscale Systems, Jülich, Germany.
[5] Institute for Biological Information Processing (IBI-6): Cellular Structural Biology, Jülich, Germany.
[6] Structural and Computational Biology Unit, European Molecular Biology Laboratory (EMBL), Heidelberg, Germany
[7] Department of Biology, Heinrich Heine University, Universitätsstr. 1, Düsseldorf, Germany.

*Corresponding authors: ivan.lazic@thermofisher.com (I.L.); c.sachse@fz-juelich.de (C.S.)

Supplementary Tables:

Supplementary Table 1. Extended imaging parameters at 300kV (wavelength λ =1.969 pm and condenser system spherical aberration C_S = 2.7 mm). Related to Table 1.

Convergence semi-angle (CSA) α [mrad]	Experimental STEM resolution based on Au measurement [Å]	Maximal STEM resolution $\lambda/(2\alpha)$ [Å]	Required pixel size at maximum STEM resolution [Å]	Depth of focus $2\lambda/\alpha^2$ [nm]
2.0	5.6	4.9	2.4	985
3.0	3.8	3.3	1.6	438
3.5	3.3	2.9	1.4	321
4.0	2.9	2.5	1.2	246
4.5	2.5	2.2	1.1	194
5.0	2.3	2.0	1.0	158
6.0	1.8	1.6	0.8	109
7.0	1.6	1.4	0.7	80
Values above are valid for spherical aberration non-corrected probes up to C_S = 2.7 mm				
Critical maximal convergence semi-angle after which probe deteriorates rapidly in all three directions is given with $\alpha_{max} = 1.34\,\lambda/(C_S\lambda^3)^{1/4}$, according to theoretical considerations[52], Sec. 3.5.1) $\alpha_{max} = 7.0\,mrad$				
Values below are well-defined and valid for aberration corrected probe				
8.0	1.4	1.2	0.6	62
9.0	1.3	1.1	0.5	49
10	1.2	1.0	0.5	39
20	0.6	0.5	0.2	10
30	0.4	0.3	0.1	4

Supplementary Table 2. Reported TMV structures based on different CTEM data sets with reported resolution and Guinier (map) B-factors.

Method and detector	Resolution [Å]	B-factor [$Å^2$]
CTEM on KODAK SO-163 film (Sachse et al. 2007[30])	4.7	280
CTEM on KODAK SO-163 film (Ge and Zhou 2011[62])	3.3	240
CTEM on Falcon II (Fromm et al. 2015[45])	3.3	150
CTEM on Falcon III (Song et al. 2019[63])	2.3	105
CTEM on K2 (Weis et al., 2019[34])	1.9	40

Supplementary Table 3. Scanning times with a total electron dose of 35 $e^-/Å^2$ using 4 pA beam current.

CSA [mrad]	2.0	3.0	3.5	4.0	4.5
Pixel size [Å]	1.70	1.23	0.98	0.98	0.75
Dwell time [µs]	4.05	2.12	1.35	1.35	0.79
Frame time for 4096 x 4096 image [s]	67.9	35.6	22.6	22.6	13.2

Supplementary Figures:

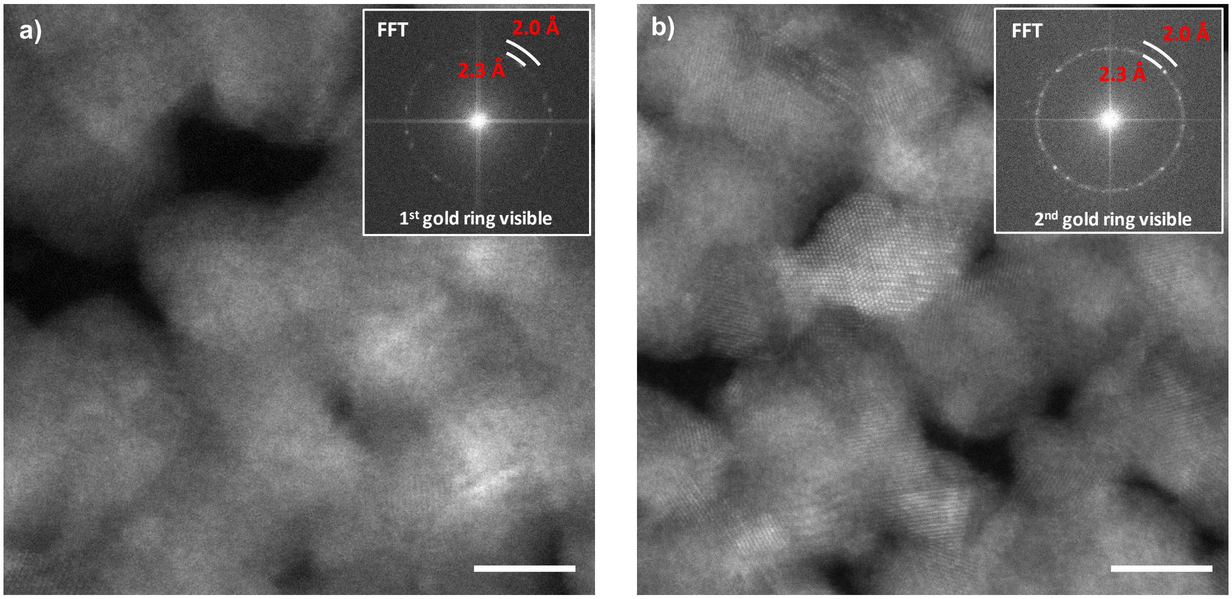

Supplementary Figure 1. Standard experimental resolution test on gold grating using ADF-STEM. (**a**) ADF-STEM image acquired with 5.0 mrad CSA beam (scale bar: 50 nm), one out of 10 typical micrographs. Power spectrum (inset) of the image shows the first gold ring corresponding to a resolution of 2.3 Å. The second gold ring is not visible at a 5.0 mrad CSA beam due to the CTF. (**b**) The same as (**a**) taken with CSA of 6.0 mrad, showing the presence of a second gold ring at a resolution of 2.0 Å. High spatial STEM resolution is confirmed on standardized gold-on-carbon sample for high-dose imaging conditions (>10^4 $e^-/Å^2$).

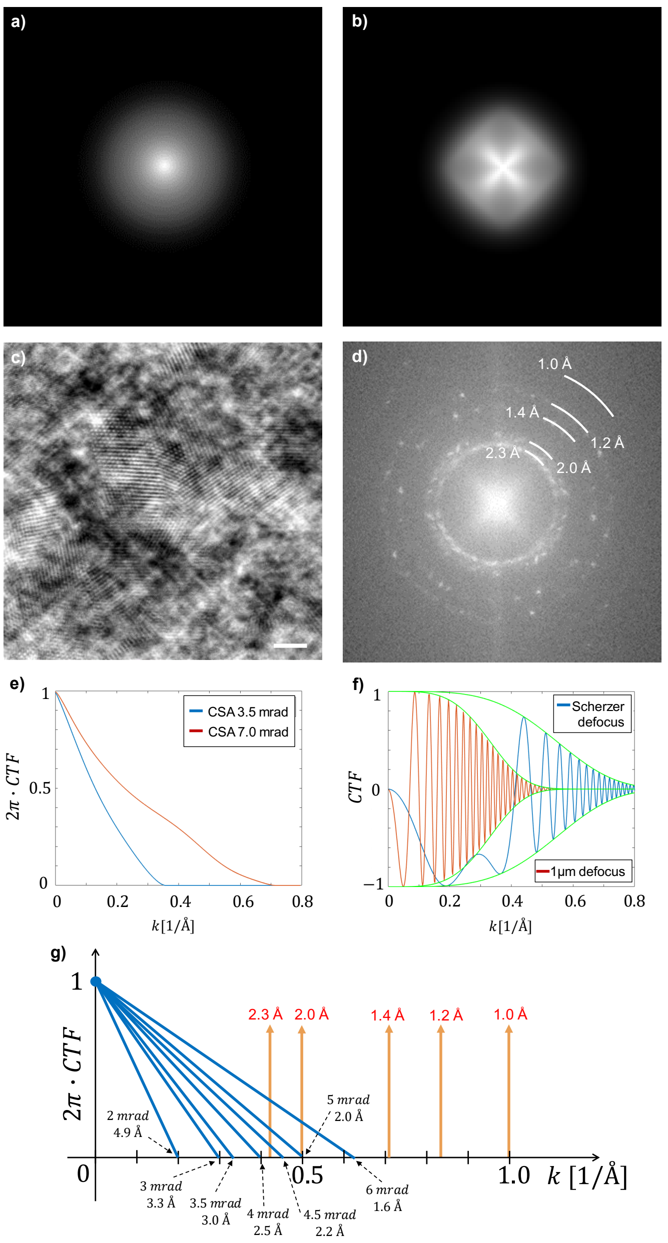

Supplementary Figure 2. Theoretical 2D CTFs of ideal integrated center of mass (iCOM)-STEM vs. iDPC-STEM using four-quadrant detector.

(**a**) Theoretical 2D rotationally symmetric CTF of iDPC-STEM based on an ideal COM detector (iCOM-STEM). (**b**) Theoretical 2D CTF of the iDPC-STEM using a four-quadrant detector, reflecting the four-fold symmetry of the detector[7,8]. (**c**) Example of iDPC-STEM image of gold on amorphous carbon sample using a four-quadrant detector at 300 kV (CSA: 20 mrad, C_s-corrected, dose: 10^4 $e^-/Å^2$, scale bar: 15 nm, one out of ~100 micrographs acquired). (**d**) Power spectrum of iDPC-STEM with characteristic gold rings of corresponding gold planes (distances indicated). Note, the four-fold cross reflection of the CTF in the center and the absence of Thon rings. (**e**) Azimuthally averaged theoretical CTF of iDPC-STEM in focus[7,8] for probe non-corrected system with 2.7 mm spherical aberration at CSA of 3.5 mrad and CSA of 7.0 mrad (Supplementary Table 1) as a function of reciprocal resolution. (**f**) Azimuthally averaged theoretical CTF of CTEM at Scherzer defocus and at 1 µm underfocus including incoherence envelopes[77]. (**g**) Simplified azimuthally averaged CTF (illustrated as straight lines) of all CSA beams used in this work with respect to gold ring positions in reciprocal space (yellow arrows).

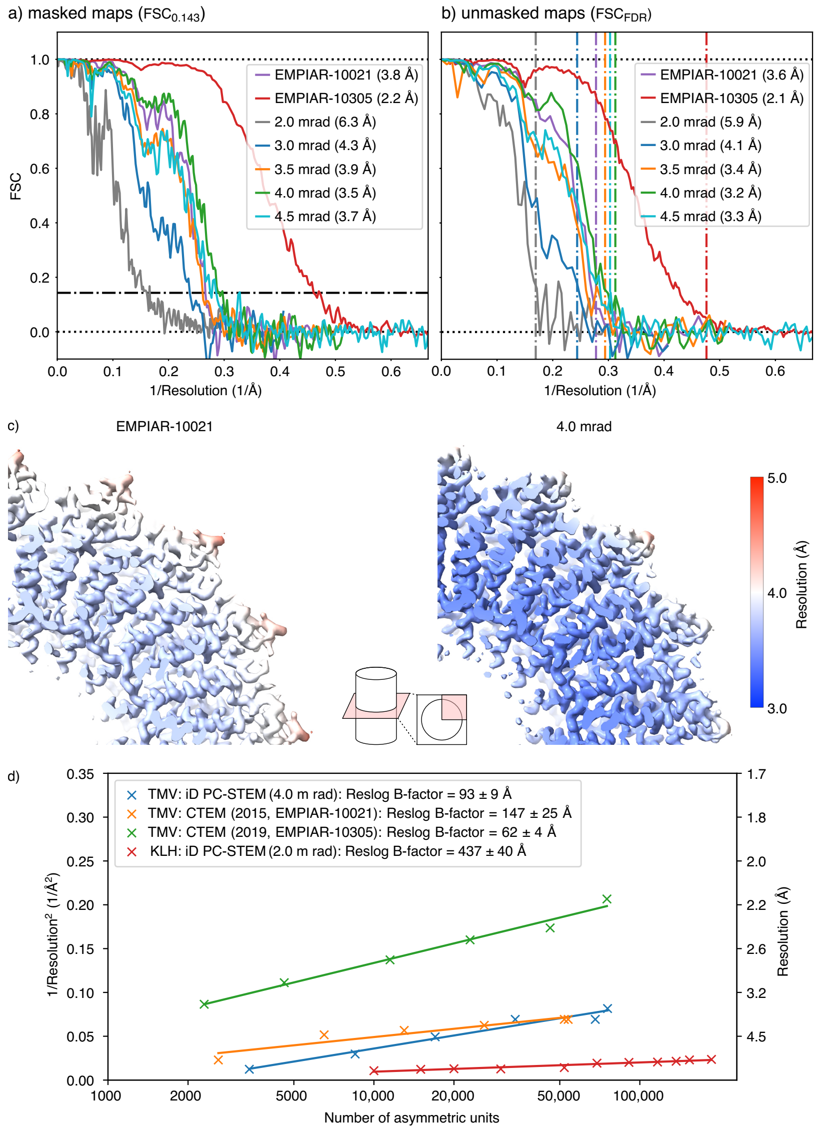

Supplementary Figure 3. Quantitative comparison of CTEM (EMPIAR-10021 and EMPIAR-10305) and iDPC-STEM data sets of different CSA beams including Fourier shell correlations (FSC), local resolution assessment and B-factor estimation.

(**a**) Resolution estimation by FSC including mask deconvolution using the 0.143 criterion[47,48]. (**b**) Resolution estimation by the mask-free FDR-FSC criterion[49]. (**c**) Color-coded local resolution estimates based on FDR-FSC[49] superimposed on molecular density of CTEM EMPIAR-10021 structure (left) and iDPC-STEM structure (right) acquired using CSA of 4.0 mrad. The best resolution of the compared cryo-EM maps is obtained for the iDPC-STEM structure acquired at 4.0 mrad CSA beam. (**d**) B-factor estimation for respective TMV and KLH data sets by a logarithmic fit of the 1/Resolution^2 as a function of Number of asymmetric units. Standard deviation of the B-factor is calculated from the covariance matrix of the fit.

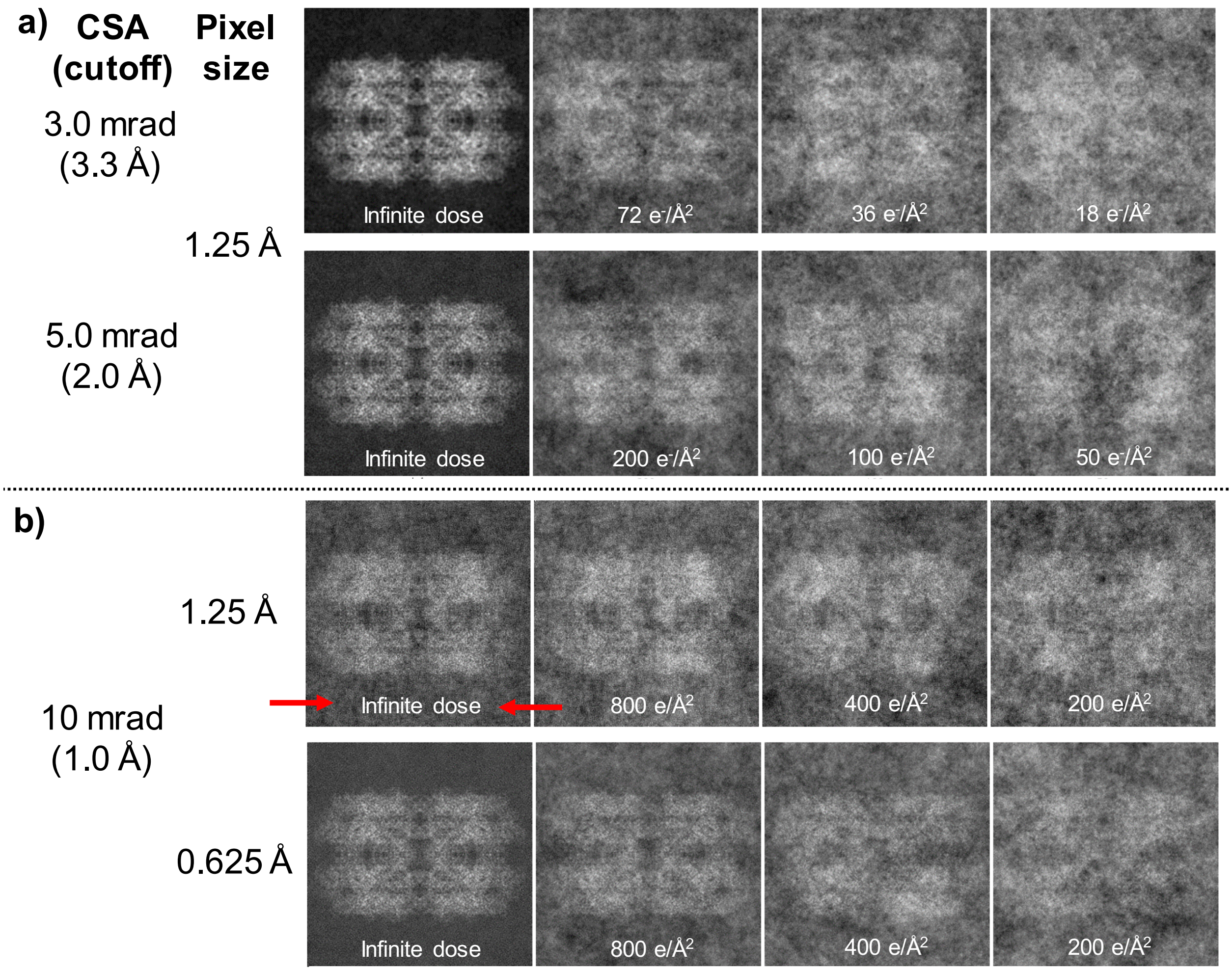

Supplementary Figure 4. Simulation of iDPC-STEM micrographs of a hemoglobin particle in amorphous ice to illustrate additional limiting effects of larger CSA beam sizes.

Imaging conditions used for simulations: voltage 300 kV, image size of 256 x 256 in (**a**) and (**b**) top row, giving pixel size of 1.25 Å, image size of 512 x 512 in (**b**) bottom row, giving pixel size of 0.625 Å. The CSAs of the beams with corresponding cutoff frequency resolutions and pixel size are indicated on the left. (**a**) Upon increase in CSA at a constant pixel size, the SNR deteriorates due to larger solid angle is covered using the same number of electrons. To maintain the SNRs, higher electron doses can be employed (follow top to bottom). (**b**) Upon increase in CSA at a constant pixel size, the cutoff frequency resolution becomes smaller than the pixel size (here 1.0 Å < 1.25Å at 10 mrad CSA is shown) causing aliasing when frequencies higher than the cut-off frequency fold back into the low-frequency signal (top). The effect of aliasing is most clearly visible at infinite dose (indicated by red arrows). The effect is not present when pixel size smaller than the cut-off resolution is used (bottom).

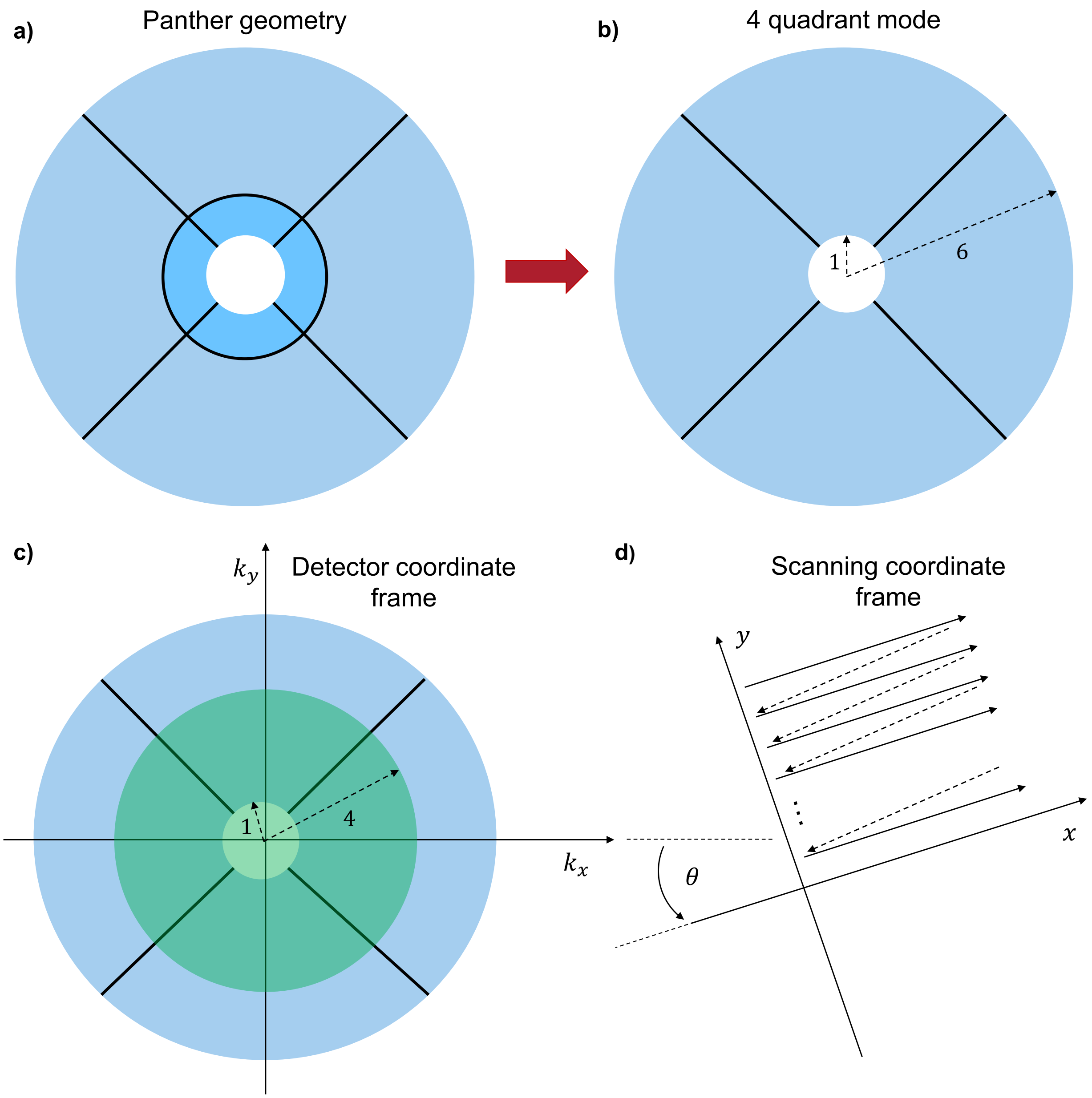

Supplementary Figure 5. Detector geometry and free scan-rotation configuration.
(a) Panther detector layout consisting of 8 segments with central hole. (**b)** Hard-wired mode represents the effective 4-segment configuration. The central hole size corresponds to 1/6 of the detector diameter **(c)** Size of the beam at the detector (approx. 2/3 of the detector), that is Bright field (BF) region or BF-disk, is also indicated with applied radius 4 times larger than the radius of the central hole. **(d)** Pattern of the scanning engine with the related detector coordinate frame. The angle (θ) determines the scan direction with respect to the detector segments and can assume any value. Signals from the opposite quadrants can be simply subtracted only when $\theta = 0$. In all other cases, the rotation matrix is applied to determine vector components in scanning frame. This is automatically considered after initial alignment, enabling user the free-scan rotation with respect to the sample.

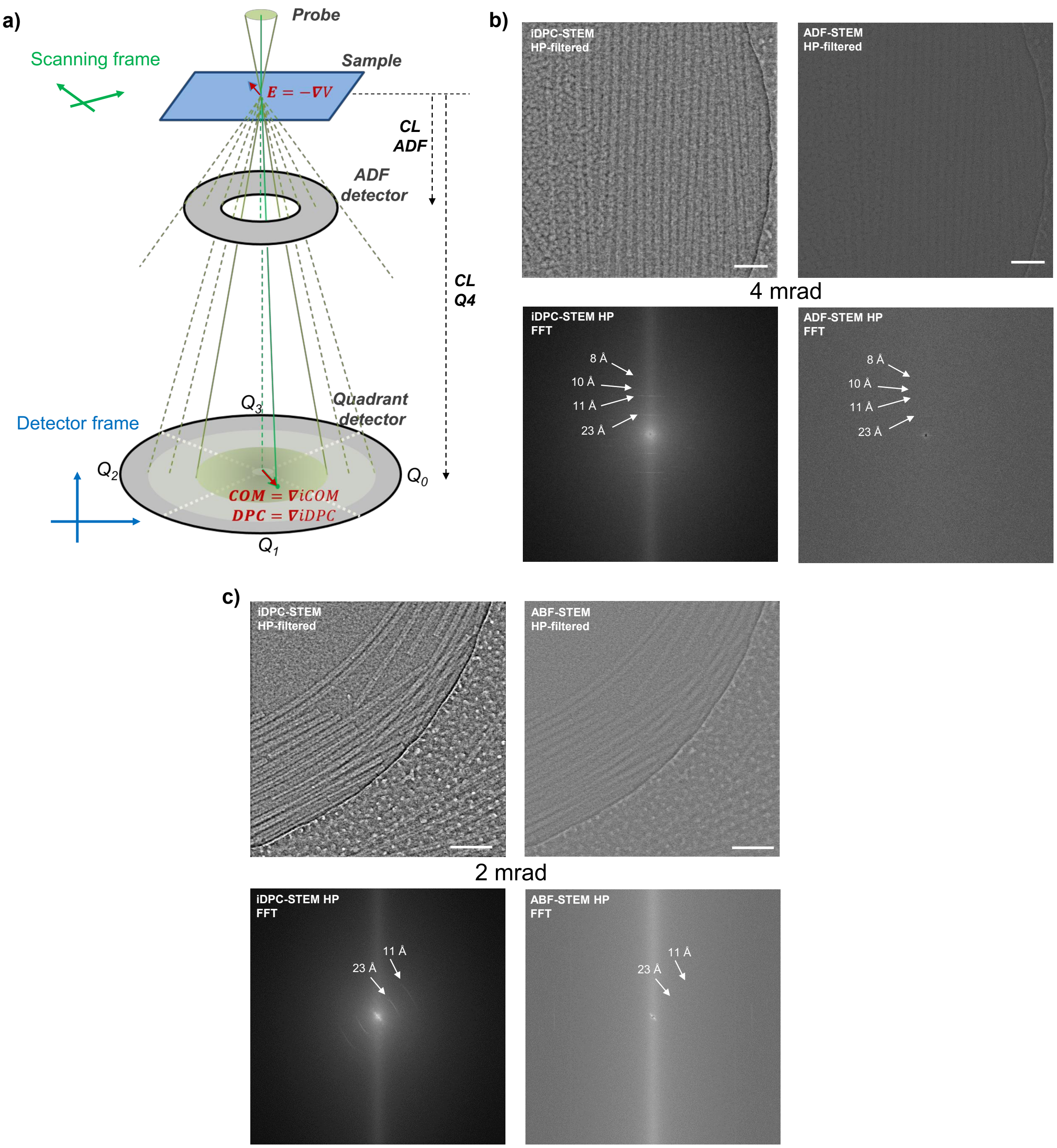

Supplementary Figure 6. Schematic on sample detector geometry and comparison between simultaneously acquired micrographs in iDPC and ADF STEM modes.
(**a**) STEM configuration schematic[9] including detector positions with respect to the sample and indicated camera lengths (CL) for ADF and 4-quadrant detector. **(b)** Preprocessed iDPC-STEM vs. ADF-STEM micrographs acquired simultaneously (one out of 20 typical micrographs) using configuration shown in **(a)**, scale bar is 100 nm. The presence of TMV layer lines (white arrows) in the Fourier transforms of the micrographs indicate improved higher resolution transfer in iDPC-STEM mode over ADF-STEM. **(c)** Preprocessed iDPC-STEM vs. ABF-STEM micrographs acquired simultaneously (one out of 20 typical micrographs). ABF-STEM is formed by summing up the 4-quadrants signals to total annular ring signal scale bar is 100 nm. Corresponding TMV layer lines in the Fourier transforms of the images are indicated. Note the improved information transfer in iDPC-STEM over ABF-STEM micrographs.
