# Supplementary material for: Single-particle cryo-EM structures from iDPC–STEM at near-atomic resolution
Source: Nat Methods. 2022 Sep 5;19(9):1126–36. doi: 10.1038/s41592-022-01586-0 (PMC9467914; doi:10.1038/s41592-022-01586-0)
Supplement: Supplementary file 2 — Reporting Summary [file 41592_2022_1586_MOESM2_ESM.pdf]

nature research

Corresponding author(s): Lazić, Sachse

Last updated by author(s): Apr 30, 2022

Reporting Summary

Nature Research wishes to improve the reproducibility of the work that we publish. This form provides structure for consistency and transparency in reporting. For further information on Nature Research policies, see our Editorial Policies and the Editorial Policy Checklist.

Statistics

For all statistical analyses, confirm that the following items are present in the figure legend, table legend, main text, or Methods section.

n/a	Confirmed	
☐	☒	The exact sample size (*n*) for each experimental group/condition, given as a discrete number and unit of measurement
☐	☒	A statement on whether measurements were taken from distinct samples or whether the same sample was measured repeatedly
☒	☐	The statistical test(s) used AND whether they are one- or two-sided<br>*Only common tests should be described solely by name; describe more complex techniques in the Methods section.*
☒	☐	A description of all covariates tested
☒	☐	A description of any assumptions or corrections, such as tests of normality and adjustment for multiple comparisons
☐	☒	A full description of the statistical parameters including central tendency (e.g. means) or other basic estimates (e.g. regression coefficient) AND variation (e.g. standard deviation) or associated estimates of uncertainty (e.g. confidence intervals)
☒	☐	For null hypothesis testing, the test statistic (e.g. *F*, *t*, *r*) with confidence intervals, effect sizes, degrees of freedom and *P* value noted<br>*Give P values as exact values whenever suitable.*
☒	☐	For Bayesian analysis, information on the choice of priors and Markov chain Monte Carlo settings
☒	☐	For hierarchical and complex designs, identification of the appropriate level for tests and full reporting of outcomes
☒	☐	Estimates of effect sizes (e.g. Cohen's *d*, Pearson's *r*), indicating how they were calculated

Our web collection on statistics for biologists contains articles on many of the points above.

Software and code

Policy information about availability of computer code

Data collection	Velox (ThermoFisher)v3.2, MAPS (ThermoFisher)v3.16
Data analysis	EMAN2 2.91, SPRING 0.86.1661, RELION 3.1, CryoSPARC 3.2, UCSF Chimera 1.15, UCSF Chimera X 1.2, Coot

For manuscripts utilizing custom algorithms or software that are central to the research but not yet described in published literature, software must be made available to editors and reviewers. We strongly encourage code deposition in a community repository (e.g. GitHub). See the Nature Research guidelines for submitting code & software for further information.

Data

Policy information about availability of data

All manuscripts must include a data availability statement. This statement should provide the following information, where applicable:

- Accession codes, unique identifiers, or web links for publicly available datasets
- A list of figures that have associated raw data
- A description of any restrictions on data availability

The following publicly available data were used in the manuscript: for TMV, 2015/2019 CTEM data sets (EMPIAR-10021 and EMPIAR-10305) and PDB coordinates of (PDB-ID 4UDV and 6SAG); for KLH, we docked the available structure PDB-ID 4BED into the iDPC-STEM map.

All data needed to evaluate the conclusions in the paper are presented in the paper and/or the Supplementary Materials. KLH iDPC-STEM (3.5 mrad) and TMV iDPC-STEM (4.0 mrad) are available as EMPIAR-11034 and EMPIAR-11042 data sets, respectively. KLH iDPC-STEM map was deposited at the EMDB (EMD-14407). The EMDB accession numbers for the reconstructed TMV cryo-EM maps including fitted PDB coordinates are EMD-13778/PDB-ID 7Q22 (CSA:2.0), EMD-13779/PDB-ID 7Q23 (CSA: 3.0), EMD-13780/PDB-ID 7Q2A (CSA: 3.5), EMD-13781/PDB-ID 7Q2R (CSA: 4.0) and EMD-13782/PDB-ID 7Q2S (CSA: 4.5).

Field-specific reporting

Please select the one below that is the best fit for your research. If you are not sure, read the appropriate sections before making your selection.

☒ Life sciences ☐ Behavioural & social sciences ☐ Ecological, evolutionary & environmental sciences

For a reference copy of the document with all sections, see nature.com/documents/nr-reporting-summary-flat.pdf

Life sciences study design

All studies must disclose on these points even when the disclosure is negative.

Sample size	Two different biological specimens were imaged, KLH and TMV. For KLH, two data sets of 760 and 687 micrographs (2.0 and 3.5 mrad) and for TMV, a total of 5 different data sets were acquired at convergence semi-angles (CSA) of 2.0, 3.0, 3.5, 4.0 and 4.5 with a total of 20, 13, 15, 20 and 28 micrographs. The number of micrographs was determined by the number of extractable particles for KLH or segments for TMV for further image processing.
Data exclusions	Micrographs of poor particle coverage and ice quality were discarded.
Replication	Due to the time-consuming nature of image acquisition and the limited access to this specialized microscope equipment (Krios and STEM unit), exact replicates were not performed.
Randomization	Randomization was not applicable in the current study because of the time-consuming nature of image acquisition and the limited access to this specialized microscope equipment (Krios and STEM unit).
Blinding	Blinding experiments were not applicable to the current study because of the time-consuming nature of image acquisition and the limited access to this specialized microscope equipment (Krios and STEM unit).

Reporting for specific materials, systems and methods

We require information from authors about some types of materials, experimental systems and methods used in many studies. Here, indicate whether each material, system or method listed is relevant to your study. If you are not sure if a list item applies to your research, read the appropriate section before selecting a response.

Materials & experimental systems

n/a	Involved in the study	
☒	☐	Antibodies
☒	☐	Eukaryotic cell lines
☒	☐	Palaeontology and archaeology
☒	☐	Animals and other organisms
☒	☐	Human research participants
☒	☐	Clinical data
☒	☐	Dual use research of concern

Methods

n/a	Involved in the study	
☒	☐	ChIP-seq
☒	☐	Flow cytometry
☒	☐	MRI-based neuroimaging
